# Vom Paradox zur Resilienz in der Krise: Ein Modell für erfolgreiches Krisenmanagement

**DOI:** 10.1007/s11612-021-00601-w

**Published:** 2021-10-05

**Authors:** Jennifer L. Sparr

**Affiliations:** ZHAW School of Management and Law, 8401 Winterthur, Schweiz

**Keywords:** Krisenmanagement, Paradox, COVID-19 Pandemie, Paradoxes Mindset, Sinnfinden und Sinnstiften, Kreativität, Organisationales Lernen, Crisis management, Paradox, COVID-19 pandemic, Paradox mindset, Sensemaking and sensegiving, Creativity, Organizational learning

## Abstract

In diesem konzeptuellen Beitrag für die Zeitschrift Gruppe. Interaktion. Organisation. wird erfolgreiches Krisenmanagement als Weg von paradoxen Spannungen in der Krise zur Resilienz der Organisation beschrieben. Widersprüchliche und doch miteinander verbundene Interessen, Bedürfnisse und Anforderungen in der aktuellen COVID-19 Pandemie dienen als Beispiele. Das Modell betont die Rolle eines paradoxen Mindsets, welches Führungskräften, Entscheidern und Betroffenen ermöglicht, paradoxe Spannungen als solche zu erkennen, anzunehmen und die Chancen im „sowohl-als auch“ (im Gegensatz zu „entweder-oder“) zu finden. Das paradoxe Mindset fördert die Auseinandersetzung mit den Spannungen in einem wiederkehrenden Prozess des Sinnfindens und Sinnstiftens. Dieser Prozess stößt im Austausch mit den unterschiedlichen Stakeholdern die Entwicklung gemeinsamer „sowohl-als auch“ Denkmodelle, kreativer Herangehensweisen und schrittweisen Lernens an. Somit stärkt die paradoxe Sichtweise die Fähigkeit von Organisationen konstruktiv mit Herausforderungen umzugehen und diese in Chancen zu verwandeln – es macht sie resilienter. Der Beitrag schließt mit drei zusammenfassenden Empfehlungen für das Krisenmanagement.

## Abstract (en)

In this conceptual contribution, successful crisis management is described as the journey from paradoxical tensions in the crisis to resilience in the organization. Contradictory, yet interrelated interests, needs and demands in the current COVID-19 pandemic are used for illustration. The developed model emphasizes the importance of a paradox mindset for leaders, decision makers and people concerned to recognize and accept paradoxical tensions in the crisis and to realize chances in a “both-and” approach (as opposed to “either-or”). The paradox mindset facilitates the reoccurring process of sensemaking and sensegiving about these tensions. This process enables the development of collective “both-and” mindsets, creative approaches, and iterative learning in the crisis. Thus, the paradox perspective enables organizations to deal constructively with tensions and to transform them into chances—it fosters the organizations’ resilience. At the end, the model is summarized in three recommendations for successful crisis management.

## Einleitung

### Paradoxe Spannungsfelder in der Krise

Krisen sind unerwartete, kritische Ereignisse, die potenziell bedrohliche Konsequenzen für Organisationen haben (Bundy et al. 2017). Eine Krise ist dadurch gekennzeichnet, dass die bisherigen Bewältigungsstrategien im Umgang mit auftretenden Problemen nicht mehr ausreichen um diese zu lösen. Eine Krise stellt damit grundsätzliche Annahmen der Organisation in Frage und ist oftmals der Startpunkt für weitreichende Veränderungen (vgl. Barnett und Pratt 2000). Die aktuelle COVID-19 Pandemie ist ein eindrückliches Beispiel einer Krise, vor deren Konsequenzen derzeit kaum eine Organisation verschont bleibt. Innerhalb kürzester Zeit konnten wir beispielweise Veränderungen in der Arbeitsorganisation beobachten, wie die Einführung von Homeoffice und virtueller Teamarbeit (vgl. Kniffin et al. 2021), aber auch kurzfristige Veränderungen im Geschäftsmodell von Unternehmen, beispielsweise die Produktion von Desinfektionsmitteln in Brauereien, Abhol- und Lieferangebote von Restaurants oder die Bereitstellung von Quarantänequartieren in Hotels.

Durch diese abrupten Veränderungen, die bestehende Arbeitsweisen, gewohnte Denkmuster und Herangehensweisen in Frage stellen, verstärken Krisen bestehende Spannungsfelder in der Organisation und fördern weitere zutage. Beispielsweise haben sich viele Führungskräfte in der Umstellung auf Homeoffice gefragt, wie sie Struktur und Kontrolle mit Flexibilität und Entscheidungsfreiheit so verbinden können, dass ihre Teams auch unter der neuen, unsicheren und sich immer wieder verändernden Situation arbeitsfähig bleiben (vgl. Kniffin et al. 2021). Einseitige Lösungen, wie beispielsweise die Überwachung durch elektronische Lösungen oder die vollständige Delegation der Arbeitsorganisation an die Mitarbeitenden führen durch ihre negativen Effekte nicht zum gewünschten Erfolg. Neue Wege der Zusammenarbeit sind nötig, die beides vereinen.

Die Notwendigkeit des „sowohl-als auch“ ist ein Hinweis darauf, dass es sich um eine „paradoxe“ Spannung handelt, das heißt sie entsteht zwischen (scheinbar) widersprüchlichen und dennoch dauerhaft miteinander verbundenen Elementen (Smith und Lewis 2011; Schad et al. 2016). Je mehr man versucht, die widersprüchlichen Elemente voneinander zu trennen, umso stärker spürt man die Spannung, die sie verbindet. Diese Elemente können gegensätzliche und doch dauerhaft miteinander verbundene Anforderungen (z. B. Kontrolle und Flexibilität), Ziele (z. B. kurzfristig wirksame und langfristig nachhaltige Lösungen) oder Bedürfnisse sein. Beispielsweise verstärkt die notwendige physische Distanz in der Pandemie das Bedürfnis nach sozialer Nähe und es scheint auf den ersten Blick schwierig bis unmöglich, beides miteinander zu vereinbaren. Für die Gesundheit und Produktivität der Menschen gehört in der Pandemie jedoch beides zusammen und bedarf neuer Ansätze, die beides ermöglichen, wie die aktuelle Homeoffice Diskussion zeigt (z. B. Turits 2021). Die Paradox-Theorie bietet einen hilfreichen Erklärungsrahmen dazu, wie Leute Spannungen wahrnehmen, sie kognitiv und emotional einordnen und konstruktiv mit ihnen umgehen können (Schad et al. 2016; Smith und Lewis 2011). Daher kann der paradoxe Blickwinkel im Krisenmanagement maßgeblich dazu beitragen, wirksame Ansätze zu finden.

### Krisen als Bedrohung und Chance

Krisenmanagement dient dem Zweck, potenziell negative Auswirkungen der Krise abzuwenden oder zu mildern und zu einem geregelten Arbeitsmodus zurück zu finden. Die Ableitung geeigneter Maßnahmen ist oft komplex, da Krisen eine Entwicklung nehmen, die schlecht vorhersehbar ist, rasch fortschreitet und aus einem Zusammenspiel von vielen Faktoren besteht. Die Aufgabe von Führungskräften in Organisationen besteht darin, die Krise zu verstehen und zu beurteilen. Dadurch können sie im Krisenverlauf kritische Entscheidungen treffen und anpassen, sowie diese erklären um die notwendige Unterstützung für Maßnahmen und Veränderungen zu sichern (Bundy et al. 2017). In dieser Komplexität, Unsicherheit und dynamischen Veränderung ist Krisenmanagement unweigerlich mit einer Vielzahl von widersprüchlichen oder als widersprüchlich empfundenen Anforderungen konfrontiert (z. B. Giustiniano et al. 2020; Pradies et al. 2021; Sarkar und Osiyevskyy 2018). Typischerweise steht dabei die von Krisen ausgehende Bedrohung im Vordergrund; nur selten werden Führungskräfte bzw. Betroffene im Allgemeinen dazu ermutigt, Krisen auch als Gelegenheiten zu begreifen (James et al. 2011; Schad et al. 2016). Möglicherweise ist dies ein Grund dafür, dass Krisenmanagement in der Management- und Organisationstheorie bislang nur eine untergeordnete Rolle spielt (Williams et al. 2017).

### Ziele des Beitrags

**Figure 1:**
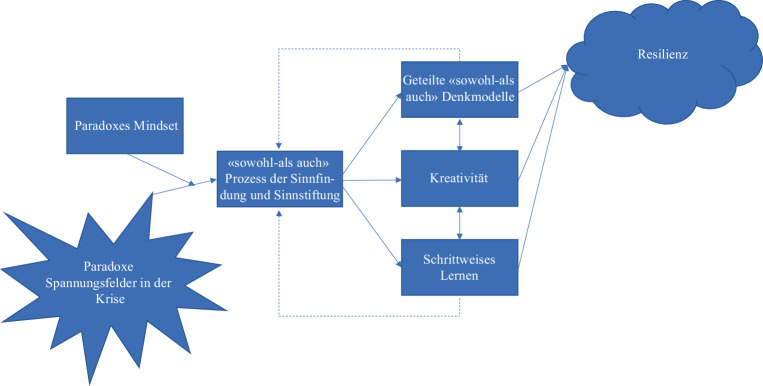

Der folgende konzeptuelle Beitrag zeigt auf, wie eine paradoxe Sichtweise dazu beiträgt, Krisen sowohl als Bedrohung als auch als Gelegenheiten zu verstehen (Schad et al. 2016), was zu erfolgreicherem Krisenmanagement und letztlich der Stärkung der organisationalen Resilienz beitragen kann. Konkret wird beschrieben, wie ein paradoxes Mindset im Krisenmanagement dabei hilft, paradoxe Spannungen aus einem „sowohl-als auch“ Blickwinkel zu verstehen und anzunehmen (Sinnfindung), sowie anderen dabei zu helfen (Sinnstiftung). Durch den akzeptierenden, differenzierenden und integrierenden Umgang mit Paradoxen entstehen geteilte „sowohl-als auch“ Denkmodelle (Miron-Spektor und Paletz 2017), Kreativität (Miron-Spektor et al. 2011, 2018) und schrittweises Lernen (Raisch et al. 2018). Die zentrale These dieses Beitrags ist, dass der „sowohl-als auch“ Ansatz über diese Mechanismen der Organisation zu mehr Resilienz verhilft (siehe Abb. 1). Der Beitrag endet mit zusammenfassenden Handlungsempfehlungen (nicht nur) für Führungskräfte.

## Paradoxes Mindset und der Prozess des Sinnfindens und Sinnstiftens in der Krise

### Ein paradoxes Mindset annehmen

Krisen können durch ihre Komplexität, die Unsicherheit und die raschen Entwicklungen überwältigend sein. Ein so genanntes paradoxes Mindset hilft Betroffenen dabei, mit dem Gefühl der Überwältigung umzugehen (Miron-Spektor und Smith 2020). Mit Mindset ist der Blickwinkel oder die Perspektive gemeint, aus welcher wir das, was wir erleben, gedanklich und gefühlsmäßig interpretieren und einordnen. Personen mit einem paradoxen Mindset nutzen die scheinbar widersprüchlichen, aber miteinander verbundenen Anforderungen um ein besseres Verständnis der Situation zu erhalten. Sie sehen den erfolgreichen Umgang mit diesen Widersprüchen als Weg zum Erfolg. Sie betrachten die Spannungen zwischen den scheinbar gegensätzlichen Elementen als Energie, welche es zu nutzen gilt und schöpfen daraus Inspiration für das „sowohl-als auch“. Als solches hilft ein paradoxes Mindset in Krisen sowohl die Herausforderungen zu sehen als auch die Gelegenheiten, die sich ergeben. Dadurch fällt es Betroffenen leichter, eine „sowohl-als auch“ Strategie zu wählen, die sowohl die Herausforderung reduziert als auch die Gelegenheiten nutzt (vgl. Miron-Spektor et al. 2018; Smith und Lewis 2011). Ein paradoxes Mindset ist also nicht etwa „paradox“ in dem Sinne, dass es selbst widersprüchlich ist, sondern bezeichnet eine positive und annehmende Haltung gegenüber paradoxen Spannungsfeldern.

Miron Spektor und Smith (2020) sprechen von der Fähigkeit, ein paradoxes Mindset anzunehmen und geben Hinweise, wie diese erlernt und verbessert werden kann. Konkret schlagen sie drei Schritte vor: (1) „Entweder/oder“ Fragen in „sowohl-als auch“ Fragen umformulieren. Beispielsweise könnte die Frage, „sollen wir physische Distanz oder soziale Nähe in Pandemiezeiten priorisieren, damit unser Team gut zusammenarbeiten kann“ (entweder/oder) positiv umformuliert werden in die Frage „wie können wir in der Pandemie die physische Distanz zur Chance für soziale Nähe machen, damit unser Team gut zusammenarbeiten kann“ (sowohl-als auch). Aus dieser positiven, die Spannung annehmenden Formulierung entstehen neue Herangehensweisen, wie beispielsweise das Ausprobieren neuer Kommunikationsformate (z. B. Nutzung von gemeinsamen virtuellen Formaten, gezieltes Aufsuchen eines informellen, bilateralen Austausches etc.). Es ist klar, dass es weiterhin ein Spannungsfeld bleibt (beispielsweise verlieren virtuelle Kaffeerunden mit der Zeit ihren Reiz) und immer wieder neue Ansätze zur Vereinbarung von physischer Distanz und sozialer Nähe gefunden werden müssen, andererseits bieten die neuen Kommunikationsformen auch neue Gelegenheiten zum Austausch mit Personen auf der ganzen Welt. Daher empfehlen die Autoren im Schritt (2) die Spannung anzunehmen und sie als einen naturgegebenen Teil der Realität anzuerkennen. Diese Akzeptanz ist wichtig, da sie ermöglicht, sich mit schwierigen Situationen besser zu fühlen. Wenn auch dies manchmal schwer fällt, empfehlen sie (3), sich von dem Problem zu distanzieren mit der Hilfe anderer einen Perspektivwechsel vorzunehmen um neue Herangehensweisen für das Paradoxe zu finden. In diese Richtung geht auch der Vorschlag von Heracleous und Robson (2020), sich von Paradoxen inspirieren zu lassen, die Wissenschaftler wie Einstein oder Bohr zu lösen hatten. In der nun schon seit mehr als einem Jahr andauernden Corona-Pandemie scheint dieser dritte Schritt besonders wichtig um immer wieder neue Chancen in den Spannungsfeldern der Krise zu finden und zu nutzen.

### Sinnfinden

Ein paradoxes Mindset schafft also einen positiven Rahmen für das Herstellen von Sinn oder kurz Sinnfinden (Sensemaking). Dabei meint Sinnfinden „einen kognitiven Prozess zur Interpretation und Integration einer Vielzahl zersplitterter, zum Teil widersprüchlicher und uneindeutiger Informationen unerwarteter Natur, die eine Verwerfung zwischen unterstellter und tatsächlicher Realität abbilden sowie die Ableitung von Handlungsentscheidungen hieraus“ (Schreyögg und Ostermann 2013, S. 130). Der „sowohl-als auch“ Sinnfindungs-Prozess besteht aus der akzeptierenden Wahrnehmung der Spannungsfelder, der Differenzierung ihrer paradoxen Elemente mit ihren Vor- und Nachteilen, welche ihre Integration unterstützt und darüber zu einer neuen Handlungsfähigkeit führt (Lüscher und Lewis 2008).

Nehmen wir das Beispiel der Führungskräfte, welche angesichts der physischen Distanz im Homeoffice einen Kontrollverlust erleben. Ein erster Impuls, enge Reporting-Strukturen einzuführen oder sogar elektronische Anwesenheitskontrollen zu installieren erweisen sich schnell als kontraproduktiv, genauso wie ein völliges Fehlen von gemeinsamen Strukturen und Regeln (entweder-oder). Die paradoxe Sichtweise hilft Führungskräften zu erkennen, dass sie ihr Bedürfnis nach Kontrolle nur dann erfüllen können, wenn sie Kontrolle an die Mitarbeitenden abgeben (vgl. Waldman und Bowen 2016) – ein Prozess, der Vertrauen erfordert. Gemeinsam mit ihrem Team kann die Führungskraft Leitlinien für die Zusammenarbeit im Homeoffice festlegen (z. B. Erreichbarkeit) und über gemeinsame Calls zu vereinbarten Zeitpunkten sicherstellen, dass sie über Arbeitsfortschritte informiert bleibt und unterstützen kann, während sie den Mitarbeitenden Flexibilität in der Gestaltung ihrer Arbeit gewährt (sowohl-als auch).

### Sinnstiften

Das paradoxe Mindset ist für alle Betroffenen in Krisen wertvoll, insbesondere jedoch für Führungskräfte. Ihnen kommt in der Vermittlung von einem Krisenverständnis eine zentrale Rolle zu, es ist ihre Aufgabe, durch geeignetes Sinnstiften (Sensegiving) ein gemeinsames Verständnis zu schaffen über das was geschieht, welche gemeinsamen Ziele verfolgt werden und wie dies geschehen soll (Foldy et al. 2008; Gioia und Chittipeddi 1991; Mumford et al. 2007). Idealerweise gelingt es ihnen, nicht nur sich selbst sondern auch den Mitarbeitenden dabei zu helfen, aus einem Zustand der Unklarheit und Verwirrung in die Arbeitsfähigkeit zu kommen (Lüscher und Lewis 2008).

Sinnstiften bezeichnet den Prozess der Einflussnahme auf das Sinnfinden anderer mit dem Ziel, dass andere ihre Wahrnehmung der Wirklichkeit auf eine vom Sender bestimmte Art und Weise definieren und sie dadurch auf eine bestimmte Art als sinnhaft erleben (vgl. Gioia und Chittipeddi 1991). Zu den wichtigsten Formen des Sinnstiftens gehört das Einordnen von Problemen und Lösungen (Foldy et al. 2008). Durch ihr eigenes „sowohl-als auch“ Sinnfinden sind Führungskräfte und Entscheider in der Lage, anderen die Sinnhaftigkeit einer „sowohl-als auch“ Betrachtung von paradoxen Spannungsfeldern zu vermitteln. Idealerweise führen sie andere durch den Prozess der akzeptierenden Wahrnehmung der Spannung, der Differenzierung zwischen den scheinbar widersprüchlichen Elementen mit ihren Vor- und Nachteilen, sowie ihrer Integration (vgl. Lüscher und Lewis 2008; Smith und Lewis 2011). Zusätzlich helfen sie durch ihre Vorbildfunktion anderen im akzeptierenden Umgang mit den Spannungen. Sie vermitteln, dass es nicht eine einzige richtige Lösung gibt, sondern dass es verschiedene Möglichkeiten geben kann, Spannungsfeldern zu begegnen und dass die Herangehensweisen immer wieder in Frage gestellt und angepasst werden können und müssen (Lüscher und Lewis 2008; Smith et al. 2016; Sparr 2018). Insgesamt sind Sinnfinden und Sinnstiften Teil eines wiederkehrenden, sozialen Prozesses, in welchem sich die Beteiligten gegenseitig beeinflussen (Gioia und Chittipeddi 1991; Kraft et al. 2018; Weick 1995).

Im Folgenden wird aufgezeigt, wie das „sowohl-als auch“ Sinnfinden und Sinnstiften drei in der Krise wichtige Prozesse anstoßen können: ein geteiltes „sowohl-als auch“ Denkmodell, Kreativität und schrittweises Lernen (siehe Abb. 1).

## Geteiltes „sowohl-als auch“ Denkmodell in der Krise

Die aktuelle COVID-19 Pandemie, ebenso wie andere Krisen unserer Zeit, beispielsweise die Finanzkrise im Jahr 2018 oder die Flüchtlingskrise der vergangenen Jahre zeigen eindrücklich die komplexe Verbundenheit von Menschen über die Grenzen von Organisationen, Gesellschaften und Staaten hinweg. In dieser Komplexität leben wir stets mit Spannungen zwischen unterschiedlichen Interessen und Perspektiven, wie beispielsweise der Spannung zwischen der individuellen Freiheit und dem kollektiven Wohlbefinden. Selten wie jetzt in der Krise ist deutlich geworden, wie sehr diese in Widerspruch stehen können und trotzdem unweigerlich miteinander verbunden sind.

Unglücklicherweise verleiten genau diese Spannungen in der Bedrohung durch die Krise gerne zu Nullsummen-Denken, bei welchem der Vorteil des einen mit dem Nachteil des anderen einhergehen muss (vgl. Van Bavel et al. 2020). Die Problematik dieses Denkens haben wir in den Diskussionen gesehen, welche beispielsweise den Schutz der Älteren in der Gesellschaft dem Recht der Kinder auf Bildung und soziale Kontakte oder wirtschaftlichen Interessen gegenüberstellten. Oftmals wird dies als „entweder-oder“ Entscheidung dargestellt, Befürworter der gegensätzlichen Positionen stellen dabei ihre Forderungen, ohne die Bedürfnisse der anderen einzubeziehen. Umso wichtiger ist es für Führungskräfte einen „sowohl-als auch“ Sinnfindungs- und Sinnstiftungsprozess anzustoßen, welcher ihnen erlaubt, die gegensätzlichen, aber miteinander verbundenen Interessen und Anforderungen als solche zu erkennen, anzunehmen und dies auch anderen zu ermöglichen um den Blick von den Bedrohungen der eigenen Interessen auf die Chancen gemeinsamer Krisenbewältigung zu lenken (vgl. Calton und Payne 2003).

Werden in diesen Prozess alle relevanten Stakeholder einbezogen, können geteilte „sowohl-als auch“ Denkmodelle entstehen. Diese entwickeln sich durch ein Teilen und Diskutieren von Vorstellungen und Ideen aus einer „sowohl-als auch“ Perspektive, welche die potenziell widersprüchlichen aber verbundenen Interessen und Bedürfnisse aller Betroffenen anerkennt (Miron-Spektor und Paletz 2017; Ashforth et al. 2014). Aus dem geteilten Verständnis heraus werden integrative Herangehensweisen möglich. Führungskräften kommt dabei eine wichtige Rolle zu – sie initiieren und moderieren den Sinnfindungs-Sinnstiftungs-Prozess, ohne ihn zu dominieren.

Ein Beispiel ist die Gründung eines „Pandemieteams“ in einem mittelständischen Industrieunternehmen bestehend aus Vertreterinnen und Vertreter der Geschäftsleitung, Bereichsleitungen, Personalleitung und Betriebsrat. Dieses Gremium diskutiert unter Einbezug ihrer unterschiedlichen Sichtweisen, Zielen und Interessen sowie unter Berücksichtigung externer Expertise (z. B. Empfehlungen des RKI oder der WHO) regelmäßig und gemeinsam über die teilweise widersprüchlichen und doch miteinander verbundenen Bedürfnisse des Unternehmens, der verschiedenen Mitarbeitendengruppen und der gesellschaftlichen Verantwortung des Unternehmens. Ziel ist es, aus einem gemeinsamen Verständnis der paradoxen Bedürfnisse „sowohl-als auch“ Maßnahmen abzuleiten und den sich verändernden Gegebenheiten in der Pandemie anzupassen. Beispielsweise muss der Schutz der Mitarbeitenden in der Pandemie gewährleistet werden, gleichzeitig aber auch die Produktionsfähigkeit des Unternehmens. Diese scheinen zunächst widersprüchlich, da die Produktionsfähigkeit durch Schutzmaßnahmen behindert scheint, und doch verbunden, da erkrankte Mitarbeitende ausfallen würden und dadurch die Produktionsfähigkeit gefährden würden. Die „sowohl-als auch“ Lösung verbindet Homeoffice und Hygienekonzept/Schichtarbeit vor Ort, welche sowohl den Schutz der Mitarbeitenden als auch die Produktionsfähigkeit gewährleisten. Gemeinsame sinnstiftende Kommunikation – persönlich und via regelmäßigen Updates aus dem Pandemieteam in der internen Mitarbeitenden-App – schafft Verständnis für die paradoxen Spannungen und Rückhalt für die getroffenen Maßnahmen in der Belegschaft (z. B. erklärt den Schutz der Mitarbeitenden und den Erhalt der Produktionsfähigkeit zu den wichtigsten, gemeinsamen Zielen in der Krise) und lädt zu kreativem, lernenden Umgang mit der Krise ein (z. B. Chancen nutzen für den Ausbau der Digitalisierung des Unternehmens).

## Kreativer Umgang mit den Spannungsfeldern in der Krise

Mit dem „sowohl-als“ Sinnfindungs- und Sinnstiftungsprozess ist der Grundstein gelegt für einen kreativen, erfinderischen Umgang mit den widersprüchlichen, aber miteinander verbundenen Interessen, Bedürfnissen und Anforderungen in der Krise. Dieser positive Zusammenhang zwischen der „sowohl-als auch“ Herangehensweise an paradoxe Anforderungen und kreativem bzw. innovativem Verhalten wurde sowohl in experimentellen Untersuchungen (Miron-Spektor et al. 2011) als auch in Feldstudien (Liu et al. 2020) nachgewiesen. Dies wird damit erklärt, dass die paradoxe Haltung eine echte Auseinandersetzung mit Komplexität fördert, ohne sich vereinfachend auf einzelne Aspekte einer Herausforderung zu konzentrieren (entweder-oder). Ähnliches kann während Krisen beobachtet werden, dass das Gefühl der Überwältigung lähmend wirkt und zu einer Konzentration auf die Kernprozesse führen kann (James et al. 2011; Moynihan 2008). Daher ist in Krisen ein paradoxes Mindset ganz besonders wertvoll um integrative Herangehensweisen für komplexe Herausforderungen zu finden und damit den Weg für neue Wege und Chancen frei zu machen (vgl. Miron-Spektor und Erez 2017; Pradies et al. 2021).

Zum Beispiel bietet der Digitalisierungsschub in der aktuellen Krise Organisationen die Chance, die digitale Zukunft in der eigenen Organisation und Branche zu gestalten. Hochschulen und andere Bildungseinrichtungen stellen dies gerade teilweise recht eindrücklich unter Beweis, indem sie nicht einfach die gewohnten Lehrformate virtuell ableisten, sondern beispielsweise multimodale und hybride Lehr-Lern-Modelle erproben (z. B. Skulmowski und Rey 2020). Durch diese Entwicklungen ist es den Universitäten möglich, ihren Leistungsauftrag zu erfüllen und gleichzeitig ihre Attraktivität durch ein innovatives Angebot zu steigern.

Dieses Beispiel zeigt auch, dass ein kreativer Umgang mit den Herausforderungen in der Krise häufig das gemeinsame Werk vieler ist. Umso wichtiger ist nicht nur das paradoxe Mindset jedes und jeder Einzelnen, sondern auch die Entwicklung eines geteilten „sowohl-als auch“ Denkmodells der unterschiedlichen Beteiligten wie oben beschrieben. Für weitere aktuelle Beispiele zum kreativen Umgang mit Paradoxen in der Krise, insbesondere mit Bezug zur COVID-19 Pandemie, verweise ich beispielsweise auf die frei verfügbare Aufsatzsammlung von Carmine et al. (2021), Keller et al. (2021), Pradies et al. (2021) und Sharma et al. (2021).

## Schrittweises Lernen

Letztendlich kann die gemeinsame, kreative Auseinandersetzung mit Paradoxen nicht nur in der bestehenden Krise zu hilfreichen „sowohl-als auch“ Ansätzen führen, sondern auch dazu beitragen, gemeinsam zu lernen um zukünftige Krisen zu verhindern oder abzumildern – ein wichtiger Aspekt und Erfolgsindikator von gelungenem Krisenmanagement. Allerdings ist Lernen in der Krise oftmals schwieriger als in Routinesituationen (Lee et al. 2020) aufgrund der hohen Tragweite und der defensiven Haltung angesichts der Unsicherheit und Bedrohung (Moynihan 2008; Boin et al., 2013). Aus der Paradox-Perspektive betrachtet, ähnelt der Prozess des Lernens einer positiven Aufwärtsspirale, welche durch den paradoxen „sowohl-als auch“ Sinnfindungs-Sinnstiftungs-Prozess angetrieben werden kann (vgl. Christianson et al. 2009). Raisch et al. (2018) beschreiben diese Spirale als eine aufeinander aufbauende Abfolge von konvergenten und divergenten Phasen, in welchen schrittweise ein besseres Verständnis der Spannungsfelder erreicht wird. Übertragen auf Lernprozesse in der Krise bedeutet das, dass in der stetigen Auseinandersetzung mit den widersprüchlichen und doch miteinander verbundenen Anforderungen (z. B. physische Distanz und soziale Nähe für erfolgreiche und sichere Teamarbeit in der Krise) ein besseres Verständnis für die einzelnen Bedürfnisse und ihre Zusammenhänge entsteht. Dadurch gelingt es zunehmend besser, ein Gleichgewicht zwischen den Bedürfnissen herzustellen. Allerdings zwingen die teils schnell und wenig planbar eintretenden Veränderungen in der Krise dazu, die Massnahmen zum Umgang mit dem Spannungsfeld laufend in Frage zu stellen und zu überprüfen. Durch eine bewusste Auseinandersetzung mit den paradoxen Anforderungen im Verlauf der Krise wird somit schrittweise ein tieferes Verständnis ihrer Komplexität ermöglicht und neue Möglichkeiten im Umgang mit ihnen entdeckt und erprobt.

Das Beispiel des oben angesprochenen Pandemieteams kann diesen Prozess verdeutlichen. Erste Maßnahmen, die zu Beginn der Pandemie rasch getroffen wurden, wie beispielsweise die Anordnung von Schichtarbeit für alle Mitarbeitenden zur Entzerrung der physischen Anwesenheit, konnte im Laufe der Zeit erprobt und durch eine geeignete Kombination von Homeoffice und Hygienevorkehrungen vor Ort so ergänzt werden, dass die Schichtarbeit nur noch in der Produktion, nicht mehr aber bei den Büroarbeitsplätzen angewendet werden musste um einen guten Schutz vor Ansteckung zu gewährleisten. Das Unternehmen war bereit, Maßnahmen gezielt auszuprobieren, anzupassen und auch zurückzunehmen, immer mit Blick auf den aktuellen Verlauf der Pandemie, den Bedürfnissen der Mitarbeitenden und des Unternehmens.

## Fazit: Vom Paradox zur Resilienz

Forschungsarbeiten zu Krisenmanagement und zu Resilienz weisen bemerkenswerte Überschneidungen auf (Williams et al. 2017). Dabei wird Resilienz als eine Eigenschaft von Individuen, Teams und Organisationen, aber auch als ein Entwicklungsprozess verstanden. Als solcher ist Resilienz eine Form des Lernens, in welchem eine Organisation sich an Widrigkeiten anpasst und dadurch Fähigkeiten erwirbt, zukünftige Herausforderungen zu bewältigen (vgl. Li 2020). Zu den wesentlichen Treibern von organisationaler Resilienz zählen die Bereitschaft, Reaktionsfähigkeit, Anpassungsfähigkeit und Fähigkeit der Organisation zu lernen (vgl. Koronis und Ponis 2018). Wie oben aufgezeigt, fördern Führungskräfte und Mitarbeitende mit einem paradoxen Mindset einen Sinnfindungs- und Sinnstiftungsprozess, welcher zu einem geteilten „sowohl-als auch“ Denkmodell führt, Kreativität anregt und schrittweise Lernprozesse in Gang setzt. Damit tragen sie vor, während und nach einer Krise dazu bei, dass die Organisation bereit und fähig ist, mit den Herausforderungen von Krisen umzugehen und nicht nur die Bedrohung, sondern auch die Chancen darin zu erkennen. Weiterhin leisten sie einen wichtigen Beitrag dazu, dass die Organisation sich kreativ und lernend an Krisensituationen anpasst, ohne ihre Stabilität zu verlieren. Abb. 1 zeigt zusammenfassend, wie Organisationen in einem wiederkehrenden Prozess des Sinnfindens- und Sinnstiftens von Spannungsfeldern in der Krise zur Resilienz finden. Sie sehen nicht nur die Bedrohung, sondern auch die Chancen (vgl. James et al., 2011; Giustiniano et al. 2020).

Die aktuelle COVID-19 Pandemie ist nicht die erste und wird bestimmt auch nicht die letzte Krise sein, die Organisationen vor Herausforderungen stellt. Folgende Empfehlungen für den Aufbau von Resilienz lassen sich aus dem entwickelten Modell ableiten:

1. *Ein paradoxes Mindset annehmen.* Nicht nur Führungskräfte profitieren davon, wenn sie sich darin üben, paradoxen Anforderungen, Zielen und Interessen nicht mit einer „entweder/oder“, sondern mit einer „sowohl-als auch“ Haltung zu begegnen. Drei Schritte können dabei helfen, nämlich die richtigen Fragen stellen („Wie kann ich beides tun?“ anstatt „Soll ich x oder y tun?“), Spannungen in ihrer Unbequemlichkeit zu akzeptieren und zu versuchen, die Herausforderungen aus einer gewissen Distanz zu betrachten (z. B. sich vorzustellen, eine Entscheidung nicht für sich selbst sondern für andere zu treffen) um neue Möglichkeiten der Herangehensweise zu entdecken (Miron-Spektor und Smith 2020). Wer wissen will, ob er bereits ein paradoxes Mindset hat, kann sich hier selbst testen: paradox.lerner.udel.edu
2. *Den Austausch mit anderen im paradoxen Sinnfindungs-Sinnstiftungs-Prozess suchen und ein gemeinsames „sowohl-als auch“ Denkmodell entwickeln.* Im Krisenmanagement kann jede und jeder einen Beitrag durch das Stellen von „sowohl-als auch“ Fragen leisten, Spannungsfelder akzeptierend thematisieren und durch das eigene Verhalten als Rollenvorbild dienen. Für eine resiliente Organisation ist es wichtig, ihre relevanten Stakeholder zu kennen und mit ihnen im offenen Austausch zu sein (siehe Beispiel Pandemieteam). Ein geteiltes „sowohl-als auch“ Denkmodell erkennt an, dass es potenziell in Konflikt stehende Interessen und Bedürfnisse gibt, die aber miteinander verbunden sind und dass daher alle mehr von einer „sowohl-als auch“ Herangehensweise anstelle von Kompromissen profitieren (Miron-Spektor und Paletz 2017). Diese Haltung gilt es zu fördern.
3. *Mit Kreativität Chancen erkennen und durch schrittweises Lernen realisieren. *Spannungsfelder und Krisen haben in all ihrer Unbequemlichkeit und Bedrohlichkeit auch immer das Potenzial, dass ein positives Neues daraus entstehen kann (z. B. Stichwort Digitalisierungsschub). Die paradoxe Haltung öffnet den Blick für einen erfinderischen Umgang mit vielfältigen Perspektiven, mit Gelegenheiten zur Erschließung neuer Ressourcen und mit der Verfolgung unterschiedlicher Strategien. Sie hilft zu erkennen und zu akzeptieren, dass Spannungsfelder zwischen verschiedenen, sich verändernden Bedürfnissen, Interessen und Anforderungen ein Teil der organisationalen Realität sind, welche Herausforderungen und Chancen mit sich bringen. Damit einher geht das Bewusstsein, dass es nicht die eine richtige Lösung gibt, die es zu finden gilt, sondern viele mögliche Wege, die es in kleinen Schritten zu erfinden, zu erproben, in Frage zu stellen und zu verwerfen gilt für ein tieferes Verständnis der Spannungsfelder und wahrhaftig neue Wege (vgl. Smith et al. 2016; Raisch et al. 2018). Organisationen sind gut darin beraten, diese schrittweisen Lernprozesse zu fördern und zu belohnen.
